# Psychologically informed patient education combined with exercise intervention is associated with improvements in tibial loading regulation, joint kinematics, and kinesiophobia in runners with medial tibial stress syndrome: a randomized controlled trial

**DOI:** 10.3389/fpsyg.2026.1880253

**Published:** 2026-07-24

**Authors:** Zeyi Zhang, Moyue Li, Ting Fan, Youping Sun

**Affiliations:** 1 College of Physical Education and Health, East China Normal University, Shanghai, China; 2 Hunan University of Medicine, Huaihua, China; 3 Institute of Physical Education, Xinjiang Normal University, Urumqi, China

**Keywords:** exercise intervention, kinesiophobia, MTSS, psychologically informed education, tibial load

## Abstract

**Objective:**

This study aimed to examine the effects and potential mechanisms of an exercise intervention (EI) combined with psychologically informed patient education (PE) on tibial loading and associated risk factors in runners with Medial Tibial Stress Syndrome (MTSS). The aim was to identify a more effective intervention approach to reduce tibial loading.

**Methods:**

A total of 104 participants were randomized to either the EI + PE group or the EI-only group (*n* = 52 per group). Ultimately, 87 participants (EI + PE: *n* = 44; EI: *n* = 43) completed the 8-week intervention and 3-month follow-up assessments and were included in the final per-protocol analysis. Tibial load and associated biomechanical risk factors were assessed at baseline, immediately post-intervention, and at follow-up, followed by further statistical analyses.

**Results:**

After 8 weeks, both EI and EI + PE groups showed significant reductions in tibial load (EI: –2.21, *p* = 0.031; EI + PE: –2.45, *p* = 0.008) and ankle plantarflexion angle (EI: –1.95, p = 0.008; EI + PE: –2.35, *p* < 0.001). At 3 months, reductions were sustained in EI, while further decreases occurred in EI + PE. Both groups showed significant increases in the estimated forces of the gluteus medius (mean change = 0.03, *p* = 0.046) and tibialis anterior (mean change = 0.10, *p* = 0.001). Only the EI + PE intervention elicited reductions in estimated soleus force (−0.23, *p* = 0.003) and Tampa Scale for kinesiophobia (TSK) scores (−7.27, *p* < 0.001).

**Conclusion:**

Among male recreational runners aged 18–30 years with a weekly running distance exceeding 20 km, EI + PE intervention was associated with reductions in tibial loading and ankle plantarflexion angle immediately after the intervention, with these parameters showing further reductions at the three-month follow-up compared with the EI group. Moreover, the EI + PE intervention was associated with increased model-derived contributions of the gluteus medius and tibialis anterior, reduced model-derived contribution of the soleus, and substantially lower TSK scores; however, whether these changes translate into clinically meaningful benefits remains to be confirmed in future studies.

## 1 Introduction

In recent years, running has emerged as one of the most widely practiced and highly popular forms of physical activity. Survey data show that participation in the Berlin Marathon increased from only 244 runners in its inaugural 1974 event (236 men and 8 women) to 40,641 participants in 2018, with male runners accounting for the majority (28,373 participants) (Reusser et al., 2021). However, behind this dramatic rise in participation lies a substantial risk of running-related injury. Franke et al. (2019) reported that the cumulative incidence of running injuries ranges from 5.6% to 14.8%, with most injuries occurring in the lower extremities—particularly the lower leg (prevalence 5.4–12.3%), knee (2.7–9.3%), and foot/toes (2.5–7.1%). When specific injury types are considered, medial tibial stress syndrome (MTSS) is especially prevalent, with an incidence as high as 9.4% (Kakouris et al., 2021). Longitudinal studies further indicate a high recurrence rate for MTSS, reaching 79.3% (Hashim et al., 2024). Recurrent MTSS episodes substantially impair exercise quality and overall quality of life in affected runners. Consequently, identifying effective interventions to reduce the risk of MTSS recurrence and promote comprehensive recovery in this population has become a pressing challenge in contemporary sports medicine.

Clarifying the pathomechanical mechanisms underlying MTSS is a prerequisite for developing effective intervention strategies. Existing evidence indicates that excessive tibial load is the most direct contributor to MTSS (Van Hooren et al., 2024). In addition, several biomechanical factors have been shown to elevate the risk of developing this condition. For example, Moen et al. (2012) reported that a greater ankle plantarflexion angle may be associated with the occurrence of MTSS, with a coefficient of 0.7 (0.5–0.9). A cohort study by Naderi et al. (2020) further demonstrated that elevated soleus activity during the push-off phase increases the risk of developing MTSS by 5%. Similarly, a systematic review by Reinking et al. (2017) revealed a significant pooled effect when greater hip external rotation was considered a risk factor for MTSS. Beyond biomechanical determinants, psychological factors also warrant attention. Because tibial pain is the primary symptom in individuals with MTSS, persistent and recurrent pain may induce fear and anxiety related to movement and movement-induced discomfort, ultimately contributing to kinesiophobia (Bingöl et al., 2025; Deshmukh and Phansopkar, 2022). This psychological condition has been linked to reductions in muscle strength, impaired postural control, and diminished proprioception during movement (Alshahrani and Reddy, 2022), thereby exacerbating existing biomechanical risk factors. For instance, Alshahrani and Reddy (2022) found that kinesiophobia reduces individuals’ positional awareness during ankle plantarflexion, which may potentially alter plantarflexion angle during running. Furthermore, Souza de Vasconcelos et al. (2023) reported that kinesiophobia also influences hip rotational patterns. Taken together, the development of MTSS appears to result from the combined influence of biomechanical and psychological factors. Therefore, intervention strategies for MTSS should address both motor control optimization and the mitigation of kinesiophobia to achieve more substantial and sustained rehabilitation outcomes.

With growing clarity regarding the mechanisms underlying MTSS, the field has increasingly turned toward exercise-based preventive strategies aimed at reducing the risk of this condition. Naderi et al. (2022) examined the effects of combining multimodal rehabilitation training with arch-support orthotic insoles on pain perception and recovery outcomes in individuals with MTSS. Their findings indicated that this approach effectively alleviated pain and symptom severity in patients with MTSS, while also producing notable improvements in physical function and perceived treatment benefit. Mendez-Rebolledo et al. (2021) implemented a 6-week neuromuscular training program in adolescent female track-and-field athletes. This program integrated jumping, landing, and running drills alongside strength, endurance, agility, balance, and core-stability exercises, with MTSS incidence monitored throughout the competitive season. The results showed that the control group experienced an MTSS incidence of 5.96 cases per 1,000 h, whereas the neuromuscular training group reported only 0.82 cases, corresponding to a relative risk of 0.17. Sharma et al. (2014) investigated the effects of gait retraining—delivered through biofeedback and/or task-specific exercises—on MTSS incidence. Compared with controls, gait retraining substantially reduced the immediate risk of MTSS, with an adjusted hazard ratio of 0.25. By week 20 of the intervention, an average of 14 individuals needed to receive gait retraining for one runner to remain free from MTSS.

It is evident that, despite the promising progress made by existing intervention studies, several notable limitations remain. First, current interventions for individuals with MTSS predominantly rely on generalized training programs that are not specifically designed based on the underlying pathomechanics of the condition. As a result, the intervention content lacks sufficient specificity, thereby constraining the potential for further improvement in treatment outcomes. Second, most existing studies have overlooked the importance of addressing kinesiophobia in individuals with MTSS, for example, by integrating psychologically informed PE into exercise-based intervention protocols. Given that the development of MTSS is driven by the combined influence of biomechanical and psychological factors, biomechanical interventions alone are unlikely to yield optimal therapeutic benefits. Consequently, integrating PE into the intervention framework has become an essential step toward enhancing the effectiveness of MTSS management—yet this dimension remains a notable gap in current research.

Therefore, this study aims to systematically investigate the effects and potential mechanisms of an EI combined with PE on tibial load and its associated risk factors in individuals with MTSS. The aim is to develop an intervention strategy that more effectively reduces tibial loading, improves movement patterns, and enhances patients’ understanding of MTSS pathophysiology and pain management.

## 2 Methods

### 2.1 Design

This study was designed as a two-arm, assessor-blinded randomized controlled trial and was approved by the Medical Ethics Committee of East China Normal University (Approval No. 2024-28). All participants provided written informed consent in accordance with the ethical principles outlined in the Declaration of Helsinki. The reporting of this study strictly adhered to the CONSORT guidelines. Participant recruitment for this study commenced on August 4, 2025, and baseline assessments were initiated on November 1, 2025.

### 2.2 Participants

Participants were recruited through community healthcare centers, social media platforms, and public advertisements. The present analysis focused on recreational runners diagnosed with MTSS. Inclusion criteria were as follows: (1) male, aged 18–30 years; (2) average weekly running distance > 20 km during the 4 weeks preceding the experiment (Zhang et al., 2025); (3) medial tibial pain induced by running or other weight-bearing activities, characterized by diffuse tenderness on palpation with a symptomatic region ≥5 cm; (4) symptom duration of at least 3 weeks; and (5) no evidence of stress fractures or other osseous pathologies on radiographs (Gomez Garcia et al., 2017). Exclusion criteria were: (1) the presence of any additional acute or chronic injuries to the trunk or lower limbs (e.g., low back pain, patellofemoral pain, or other stress fractures); and (2) inability to continuously complete running tasks during testing (Nixon et al., 2025). A total of 87 participants were included in the final analysis, and their baseline characteristics are presented in Table 1. Comparative analyses showed no statistically significant between-group differences in either the primary outcome or secondary outcomes at baseline. During the running trials, participants selected their preferred running speed within a controlled range of 2–4 m/s, while cadence was maintained at 170 ± 5% steps/min (162–179 steps/min) (Baker et al., 2025). All participants adopted a rearfoot strike pattern during the experimental trials. Furthermore, the primary analysis included recreational runners with a weekly training volume of at least 24 km (Leacox et al., 2025).

**Table 1:** Demographic and baseline characteristics of the study’s patients.

Characteristic	EI+PE group (final analysis, *n* = 44)	EI group (final analysis, *n* = 43)	*p*-value
Demographics			
Age (years)	20.47 (2.90)	19.57 (1.12)	
Height (m)	1.79 (0.05)	1.77 (0.06)	
Body mass (kg)	71.72 (9.23)	69.64 (8.19)	
Mean speed (m/s)	3.66 (0.31)	3.49 (0.41)	
Primary outcome			
Tibial load (*N*/kg)	16.33 (4.62)	16.18 (5.12)	0.88
Secondary outcomes			
Ankle plantar/dorsiflexion angle (°)	26.25 (3.58)	26.32 (3.51)	0.93
Hip in/external rotation angle (°)	22.38 (19.55)	22.74 (13.67)	0.92
Estimated gluteus medius force (*N*/kg)	0.06 (0.04)	0.07 (0.08)	0.60
Estimated gluteus minimus force (*N*/kg)	0.05 (0.04)	0.05 (0.04)	0.97
Estimated tensor fasciae latae force (*N*/kg)	0.12 (0.03)	0.12 (0.02)	0.80
Estimated soleus force (*N*/kg)	0.91 (0.42)	0.94 (0.45)	0.76
Estimated tibialis anterior force (*N*/kg)	0.49 (0.20)	0.50 (0.14)	0.77
TSK score	39.48 (6.25)	40.30 (4.87)	0.50

### 2.3 Randomization

Following the baseline assessment, participants were randomly allocated using a random-number sequence generated in Python 3.8 (Python Software Foundation, USA) with a block size of six, ensuring balanced sample sizes between the EI + PE and EI groups. To achieve allocation concealment, the randomization sequence was generated in advance by a researcher not involved in participant recruitment or intervention delivery. Group assignments were placed in sealed, opaque envelopes and were not disclosed until immediately before the initiation of the intervention.

### 2.4 Outcome measures

Before the formal experiment commenced, participants completed a subjective assessment using the TSK. The scale comprises 17 items rated on a 4-point Likert scale (1 = strongly disagree, 4 = strongly agree), yielding a total score ranging from 17 to 68. Previous studies have confirmed that the Chinese version of the TSK demonstrates good reliability and validity (Wei et al., 2015). Participants then performed approximately 15 min of static stretching and light jogging to adequately activate the trunk and lower-limb musculature and reduce initial stiffness. To minimize the potential influence of clothing variability on data acquisition accuracy, all participants were provided with standardized experimental attire, including compression tops, shorts, and running shoes. After completing the preparatory procedures, 39 reflective markers (14-mm diameter) were affixed according to the Plug-in-Gait full-body model on the head; spine (C7 and T10); pelvis (bilateral ASIS and PSIS); thighs; knees; shanks; ankles; and feet, among other standard anatomical landmarks. A static calibration trial was subsequently conducted to obtain static reference data.

After completing the static calibration, participants proceeded to the running assessment. The testing environment consisted of an indoor runway with two embedded AMTI BMS400600 force plates (Advanced Mechanical Technology Inc., USA), which recorded ground reaction forces at 1,000 Hz. To ensure that the affected limb accurately contacted the force plates, each participant adjusted their starting position based on individual stride length prior to data collection. Concurrently, a Vicon V2.2 three-dimensional motion capture system (Vicon Motion Systems Ltd., UK) equipped with 12 high-speed cameras sampled kinematic trajectories at 200 Hz. In addition, a Noraxon Ultium wireless surface EMG system (Noraxon Inc., USA) recorded muscle activity at 1,000 Hz from the gluteus medius, tensor fasciae latae, tibialis anterior, and soleus. Electrode placement followed standardized guidelines: the gluteus medius electrode was positioned at the midpoint between the iliac crest and greater trochanter; the tensor fasciae latae electrode at the proximal one-sixth between the ASIS and the lateral femoral condyle; the tibialis anterior electrode at the distal one-third between the fibular head and medial malleolus; and the soleus electrode at the distal two-thirds between the medial femoral condyle and medial malleolus. During testing, participants completed five valid running trials at a self-selected speed, with approximately 1 min of rest between trials to minimize fatigue-related confounding.

### 2.5 Data processing

#### 2.5.1 Calculation of basic parameters

This study focused exclusively on the biomechanical performance of the affected limb during the stance phase of running. Data processing began with kinematic and kinetic signals. Reflective marker trajectories and ground reaction force (GRF) data were filtered using a fourth-order zero-lag Butterworth low-pass filter with cutoff frequencies of 12 Hz and 45 Hz, respectively (Traut et al., 2024; Zandbergen et al., 2023), to minimize high-frequency noise. Pelvic and lower-limb joint angles were subsequently resolved in three dimensions using an X–Y–Z Cardan rotation sequence, with all angles reported in degrees (°) (Jandacka et al., 2023). For downstream OpenSim modeling, kinematic data were exported in *.trc* format and kinetic data in *.mot* format. EMG data were processed separately using Noraxon MR3.16 software (Noraxon Inc., USA). The processing pipeline included band-pass filtering (20–450 Hz) to remove artifacts, full-wave rectification, and smoothing with a 250-ms moving-window root mean square (RMS). Finally, peak RMS values from each trial were extracted and used to normalize the frame-by-frame EMG signals after RMS processing (Korak et al., 2018). These EMG measurements served primarily as external validation for the muscle-driven outputs generated by the OpenSim model.

#### 2.5.2 Calculation of tibial loading

Tibial compressive load is commonly defined as the axial compressive force acting on the distal tibia, which is equivalent in magnitude to the compressive force at the ankle joint (Matijevich et al., 2019; Matijevich et al., 2020). Accordingly, previous studies have indicated that $F_{\text{tibia}}$ can be estimated using a two-dimensional (sagittal-plane) lower-limb model, in which the tibial load is computed as the sum of the ankle joint reaction force induced by the GRF ( $F_{\mathrm{ext}}$ ) and the estimated compressive force generated by the plantarflexor muscles—including the soleus, medial gastrocnemius, and lateral gastrocnemius—acting on the tibia ( $F_{\mathrm{int}}$ ), while neglecting contributions from other muscles (Scott and Winter, 1990).


$$F_{\text{tibia}}(t)=F_{\mathrm{ext}}(t)+F_{\mathrm{int}}(t)$$

During the modeling process, previous studies have indicated that the mass and moment of inertia of the foot can be considered negligible (Matijevich et al., 2019; Matijevich et al., 2020). Consequently, $F_{\mathrm{ext}}$ was treated as equivalent to the GRF acting along the tibial axis. However, to better capture the heel-strike impact characteristics, the GRF signal was low-pass filtered using a cutoff frequency of 45 Hz rather than the conventional 15 Hz (Scott and Winter, 1990; Zandbergen et al., 2023).


$$F_{\mathrm{ext}}(t)=|{\underset{\mathrm{GRF}}{\to }}^{*}(t)|\cdot \cos\;\beta (t)$$

In this formulation, *β* represents the angle between the GRF vector and the tibial segment in the sagittal plane. The internal compressive force *F_int_* was computed as the ankle joint moment *M_ankle_* divided by the moment arm of the Achilles tendon (*r_at_*), which was assumed to be constant at 0.05 m in accordance with previous studies (Farris and Sawicki, 2012; Honert and Zelik, 2016; Matijevich et al., 2019; Matijevich et al., 2020).


$$F_{\mathrm{int}}(t)=\frac{M_{\text{ankle}}}{r_{\mathrm{at}}}=\frac{\mathrm{CO}P_{x,\text{ankle}}\cdot \mathrm{GR}F_{z}(t)}{0.05}$$

In this framework, *COP_x,ankle_* represents the anteroposterior position of the center of pressure relative to the ankle joint center. The ankle joint moment *M_ankle_* was then computed as the product of *COP_x,ankle_* and the vertical component of the GRF (*GRF_z_*) (Matijevich et al., 2019; Matijevich et al., 2020; Scott and Winter, 1990).

It should be noted that this model is based on several established assumptions, including the neglect of foot inertia, the use of a fixed Achilles tendon moment arm of 0.05 m, exclusion of contributions from non-plantarflexor muscles, and the simplification of a three-dimensional running task into a sagittal-plane estimate. These assumptions are consistent with modeling approaches in prior studies and facilitate the derivation of tibial compressive load estimates that are interpretable and reproducible under standardized conditions. At the same time, we recognize that the onset and progression of MTSS are likely influenced by multiple mechanical factors beyond axial compressive load; bending moments, shear forces, tibial torsion, local periosteal traction, and cumulative load exposure may all contribute to its pathomechanics. Accordingly, the findings of the present study should be interpreted within the context of this modeling framework. Future research could integrate more individualized and three-dimensional modeling approaches and incorporate multidimensional loading metrics to further advance the understanding of MTSS-related mechanical mechanisms.

#### 2.5.3 OpenSim modeling

Estimation of muscle force production during running was performed using the OpenSim 4.4 platform (Stanford University, USA). The modeling and computational pipeline comprised several key stages, including model scaling (Scale), inverse kinematics (IK), the Residual Reduction Algorithm (RRA), and Computed Muscle Control (CMC).

First, during the Scale stage, marker positions from the static calibration trial were used to match the generic musculoskeletal model to each participant’s anthropometric characteristics, thereby ensuring subject-specific accuracy in subsequent analyses (Yap et al., 2021). Second, IK was performed. Using dynamic marker trajectories collected by the motion capture system, IK computed the three-dimensional joint angles of the pelvis, hip, knee, and ankle (Yap et al., 2021). The objective of IK was to minimize the root-mean-square error between experimental and model markers, yielding joint kinematics that closely reproduced the participant’s actual movement patterns (Yap et al., 2021). After obtaining the initial joint kinematics, the RRA was applied to improve dynamic consistency. Because soft tissue motion and marker placement errors are unavoidable during experimental data collection, non-physiological residual forces and moments may appear in the dynamic equations. RRA addresses this issue by slightly adjusting the model’s segment mass properties and joint trajectories, thereby reducing residual terms and enhancing the stability and reliability of the dynamic analysis.

After completing the dynamic consistency adjustments, the pipeline proceeded to the CMC stage. This method, grounded in optimal control theory, distributes the net joint moments across the synergistic muscle groups by minimizing the sum of squared muscle activations, thereby ensuring physiologically plausible solutions. Subsequently, muscle force production during the stance phase was computed by integrating the muscle-specific mechanical properties embedded within the OpenSim model—such as maximal isometric force, as well as the force–length and force–velocity relationships (Van Hooren et al., 2024).

### 2.6 Interventions

The intervention was conducted in a university biomechanics laboratory and administered by two professional physical therapists who were native Chinese speakers. One therapist was responsible for delivering patient education, while the other supervised the exercise intervention. The patient education component employed a partially adapted standardized intervention script, similar to that described by Sheikhi et al. (2024), while allowing therapists to tailor the sessions based on participants’ pain presentation, movement-related fear, training load, and individual questions. It should be noted that participants were instructed to maintain their habitual running routines throughout the 8-week intervention period and to avoid substantial increases in running distance, training frequency, or intensity. While the EI strength-training sessions may have replaced a small portion of supplementary or strength-training activities within their usual training regimen, they were not intended to substitute for regular running sessions. The detailed intervention procedures are provided in the Supplementary material.

#### 2.6.1 Patient education

Participants in the EI + PE group received three one-on-one supervised education sessions over the 8-week intervention period (in addition to the exercise program), with each session lasting approximately 30–45 min. In this study, the patient education was guided by a psychologically informed rehabilitation framework, primarily based on the core principles of the fear-avoidance model (Vlaeyen and Linton, 2000) and pain neuroscience education (Louw et al., 2016). The first session focused on obtaining a detailed understanding of the patient’s pain characteristics, including pain intensity, pain location, situations in which symptoms were most pronounced during daily activities, the patient’s beliefs regarding the causes of pain, and their expectations for treatment (Sheikhi et al., 2024). Based on this information, the subsequent two sessions were tailored by the therapist to address individual needs.

The educational content generally included: (1) an explanation of the mechanisms and contributing factors of MTSS, such as how ankle and proximal joint movement patterns and muscle force-production strategies influence tibial loading; (2) pain-management strategies during running, including reducing excessive ankle plantarflexion and hip external rotation, maintaining proper lower-limb alignment, and adopting an appropriate shock-absorption pattern; (3) guidance on pacing and load-management strategies to appropriately modify physical activity—for example, reducing training volume or load if pain exceeds 5/10; and (4) addressing patient-specific questions raised during the sessions (Sheikhi et al., 2024). The education was delivered through verbal instruction supplemented with visual illustrations to enhance comprehension. The psychological constructs specifically targeted by the patient education component included movement-related fear, pain-related threat appraisal, and self-efficacy for symptom self-management. Specifically, movement-related fear corresponded to items 2–3 of the educational content and was assessed using TSK scores; pain-related threat appraisal corresponded to items 1 and 4, and self-efficacy corresponded to items 3–4. These latter constructs were addressed through individualized discussions, load-management strategies, and verbal feedback; however, they were not systematically evaluated using dedicated psychometric instruments.

Therapist fidelity was monitored via video recordings of the education sessions to ensure adherence to the standardized intervention protocol. Participants’ understanding of the educational content was assessed immediately after each session by asking them to verbally summarize the key points. Kinesiophobia was evaluated using the TSK, whereas pain catastrophizing was not systematically assessed.

#### 2.6.2 Exercise intervention

The exercise intervention was delivered in small-group sessions under the continuous supervision of a designated physical therapist. The EI + PE and EI groups trained on separate schedules, with the EI + PE group attending sessions on odd-numbered days and the EI group on even-numbered days. The 8-week intervention consisted of three sessions per week, each lasting approximately 60 min. The program was structured into three progressive phases: during the first 2 weeks, training emphasized motor control of the hip and ankle musculature; over the subsequent 3 weeks, motor control continued while muscle strength was progressively increased; and during the final 3 weeks, external load was incorporated through weighted exercises to further enhance training difficulty (Sheikhi et al., 2024).

### 2.7 Sample size calculation and statistical analysis

For the sample size estimation, a statistical power of 80%, a significance level (*α*) of 0.05, and a medium effect size of 0.25 were assumed, yielding a minimum requirement of 43 participants per group. All calculations were performed using GPower 3.1.9.2 (Heinrich Heine University Düsseldorf, Germany). Considering the potential attrition inherent to intervention studies, the final sample size was increased to 52 participants per group.

Statistical analyses were conducted using SPSS 26.0 (IBM Corp., Armonk, USA). The primary outcome measure was tibial load, and secondary outcomes included ankle plantar/dorsiflexion angle (°), hip in/external rotation angle (°), estimated forces of the gluteus medius (N/kg), gluteus minimus (N/kg), tensor fasciae latae (N/kg), soleus (N/kg), tibialis anterior (N/kg), and TSK score. Initially, the data were assessed for basic assumptions of normality and homogeneity of variance. When the sphericity assumption was violated, Greenhouse–Geisser corrections were applied to adjust the degrees of freedom. In cases where the homogeneity of variance assumption was not met, Welch’s correction was used for between-group comparisons. A 2 × 3 mixed-model two-way ANOVA was first employed to examine differences between groups (EI + PE vs. EI) across time (baseline, post-intervention, and 3-month follow-up). When a significant group × time interaction was detected, *post hoc* pairwise comparisons with appropriate corrections were performed across the three time points. Secondary outcomes were adjusted for multiple comparisons using the Benjamini–Hochberg false discovery rate (FDR) procedure. To mitigate the risk of false-positive findings arising from multiple comparisons among secondary outcomes, all post hoc comparisons for these outcomes were subjected to Benjamini–Hochberg FDR correction. This correction was applied exclusively to secondary outcome variables, including ankle plantarflexion/dorsiflexion angle, model-estimated soleus force, and TSK scores, etc. Following FDR correction, statistically significant results were primarily observed for ankle kinematics, model-estimated soleus force, and TSK scores, whereas all other secondary outcomes did not retain statistical significance and should therefore be interpreted cautiously as exploratory findings. To evaluate the magnitude of the observed effects, partial eta squared (ηp^2^) was calculated and interpreted using commonly adopted benchmarks: negligible (ηp^2^ < 0.01), small (0.01 ≤ ηp^2^ < 0.06), medium (0.06 ≤ ηp^2^ < 0.14), and large (ηp^2^ ≥ 0.14) (Yang et al., 2026).

## 3 Results

### 3.1 Patients

**Figure 1:**
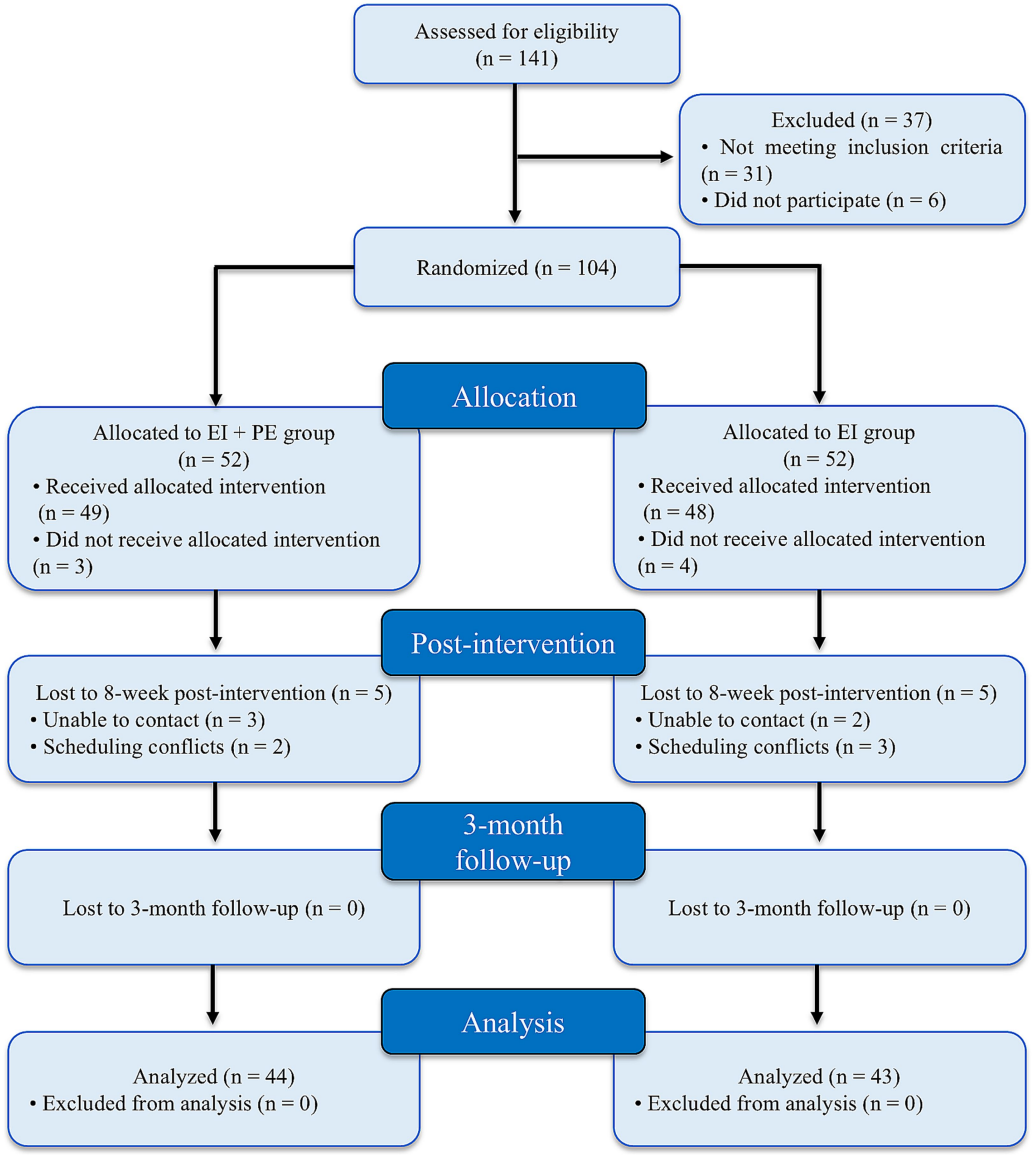
CONSORT flow diagram.

A total of 141 participants were initially screened for eligibility (Figure 1), of whom 37 were excluded. The remaining 104 participants were randomly allocated to either the EI + PE group or the EI group. Following allocation, 17 participants were excluded from the analysis (8 in the EI + PE group and 9 in the EI group). Specifically, during the intervention period, seven participants (3 in the EI + PE group and 4 in the EI group) were excluded due to non-receipt of the allocated intervention, and an additional 10 participants withdrew over the 8-week intervention period. Among these, five participants (3 in the EI + PE group and 2 in the EI group) were lost to follow-up due to inability to contact, while the remaining five (2 in the EI + PE group and 3 in the EI group) withdrew for personal reasons or due to scheduling conflicts with the intervention sessions. No further attrition occurred during the 3-month follow-up period. Importantly, no statistically significant differences were observed at baseline between completers and non-completers in either primary or secondary outcomes (*p* > 0.05). Consequently, 87 participants completed both the intervention and follow-up assessments and were included in the final analysis, including 44 participants in the EI + PE group and 43 participants in the EI group. In terms of numbers, the participants excluded from the final analysis were similar between groups, with eight in the EI + PE group and nine in the EI group, indicating a generally balanced pattern of attrition. The final analysis was conducted on a per-protocol basis, including only complete cases. In this study, we prioritized per-protocol (PP) and complete-case (CC) analyses rather than an intention-to-treat (ITT) approach for the following reasons. First, ITT analysis, by including non-adherent, partially adherent, and dropout participants within their originally assigned groups, inevitably dilutes the effective intervention dose, thereby potentially underestimating the true efficacy of the intervention under ideal implementation conditions. In contrast, PP analysis, by restricting the analysis to participants with complete exposure to the intervention protocol, more directly addresses the effectiveness question of whether the intervention is efficacious when properly implemented. This approach also improves the consistency of the analytical dataset and measurement reliability, while reducing additional uncertainty introduced by missing-data handling under ITT frameworks, such as the potential violation of assumptions underlying imputation models. Incomplete data resulting from participants who did not complete the full intervention protocol were excluded from the analysis in this study. In this study, most participants completed the scheduled exercise and education sessions. The minimum adherence threshold was defined as completion of at least 80% of the total intervention sessions, corresponding to at least 20 exercise sessions in the EI group and at least 22 total intervention sessions in the EI + PE group. After the intervention, we recorded the number of completed intervention sessions for each group. Participants in the EI group completed an average of 23.21 sessions, whereas those in the EI + PE group completed an average of 25.70 sessions. These findings indicate good intervention adherence and demonstrate that the participants met the adherence criteria established *a priori*. No adverse events were observed throughout the intervention period. Overall, participant adherence was good, and the intervention was implemented as planned. The EI + PE group had a mean age of 20.47 years, a height of 1.79 m, a body mass of 71.72 kg, and a running speed of 3.66 m/s, whereas the EI group had a mean age of 19.57 years, a height of 1.77 m, a body mass of 69.64 kg, and a running speed of 3.49 m/s (Table 1).

### 3.2 Validation of the OpenSim model

**Figure 2:**
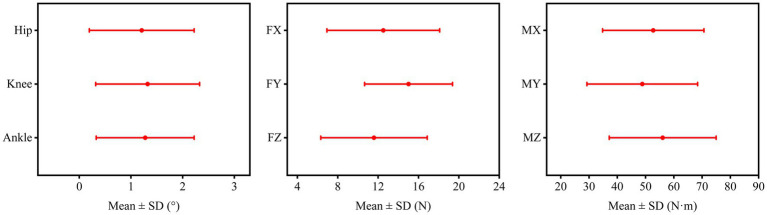
Differences between the OpenSim-simulated kinematic and kinetic data and the experimentally measured data. *Hip*, *Knee*, and *Ankle* represent the discrepancies between the experimentally measured sagittal-plane joint angles and those generated by the OpenSim model. *FX*, *FY*, and *FZ* denote the residual forces in the anteroposterior, vertical, and mediolateral directions, respectively, while *MX*, *MY*, and *MZ* correspond to the residual moments in the anteroposterior, vertical, and mediolateral directions.

**Figure 3:**
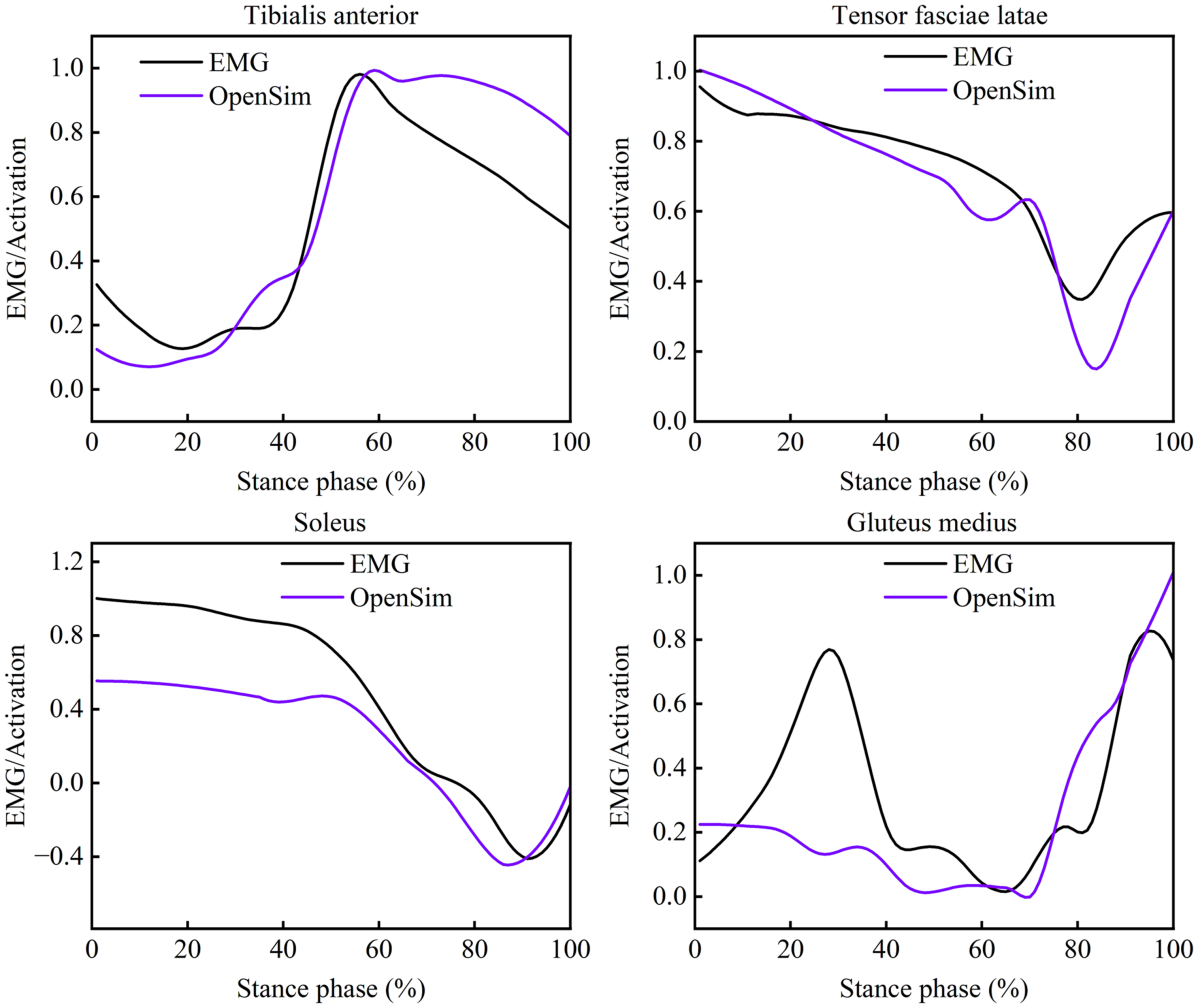
Differences between the OpenSim-simulated muscle activation profiles and the experimentally recorded EMG data.

As shown in Figures 2, 3, the differences between the OpenSim-simulated hip, knee, and ankle sagittal-plane joint angles and the experimentally measured kinematics were all less than 5°. The force and moment residuals were below 20 N and 75 N·m, respectively, and the simulated muscle activation profiles closely resembled the experimentally recorded curves. Collectively, these results indicate that the model demonstrated acceptable tracking performance and that the EMG comparison supported the plausibility of the model-derived outputs. Prior to plotting, both the EMG envelopes and model-derived outputs were subjected to normalization and baseline correction (detrending). Consequently, values below zero indicate that the signal falls beneath the baseline-corrected reference level and should not be interpreted as physiologically negative EMG activity or negative muscle activation.

### 3.3 Basic assumption testing for statistical analyses

For all outcome measures, the model residuals met the assumption of normality, as indicated by Shapiro–Wilk test *p*-values ranging from 0.330 to 0.345, confirming that the residuals adhered to model assumptions. Homogeneity of variance between groups was also confirmed at each time point, as indicated by Levene’s test *p* values ranging from 0.980 to 0.999. However, Mauchly’s test indicated violations of the sphericity assumption for several variables, including the gluteus medius and tensor fasciae latae, with *p* values ranging from <0.001 to 0.005. Therefore, Greenhouse–Geisser corrections were applied to all F tests involving Time and Group × Time effects to minimize the potential influence of sphericity violations on the repeated-measures analyses.

### 3.4 Primary outcome

For tibial loading, the two-way ANOVA revealed a significant interaction between group (EI vs. EI + PE) and time (baseline, post-intervention, and follow-up) (F_[2,170]_ = 4.83, *p* = 0.009, ηp^2^ = 0.036). *Post hoc* analyses showed that, relative to baseline, the EI group exhibited significantly lower tibial loading at both the post-intervention assessment (mean difference = −2.21; F_[2,170]_ = 3.84; *p* = 0.031; ηp^2^ = 0.057) and the follow-up assessment (mean difference = −2.60; F_[2,170]_ = 3.84; *p* = 0.011; ηp^2^ = 0.057), with no significant difference between post-intervention and follow-up values (*p* = 0.705).

**Figure 4:**
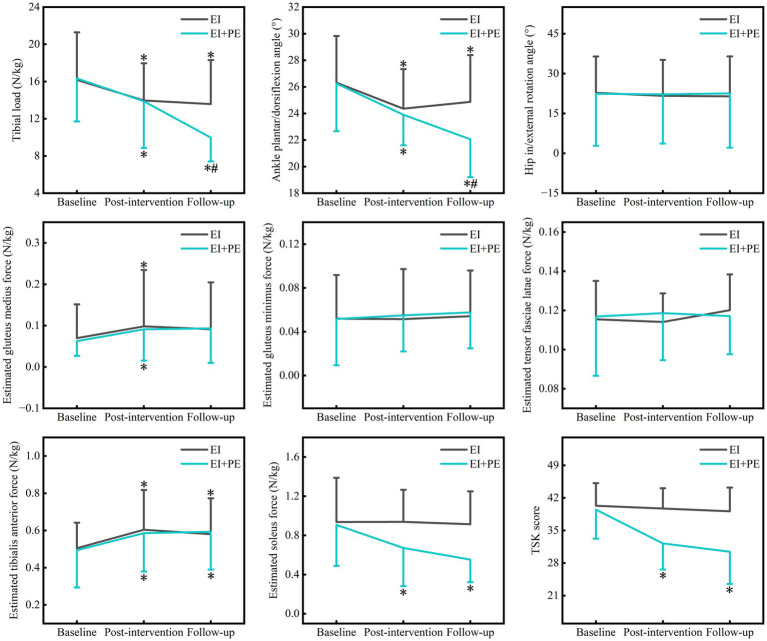
Changes in the primary and secondary outcomes for both groups at baseline, post-intervention, and follow-up assessments. *indicates a statistically significant difference compared with baseline at post-intervention or follow-up, and # indicates a statistically significant difference between post-intervention and follow-up.

In the EI + PE group, tibial loading was significantly lower than baseline at both the post-intervention assessment (mean difference = −2.45; F_[2,170]_ = 25.03; *p* = 0.008; ηp^2^ = 0.280) and the follow-up assessment (mean difference = −6.36; F_[2,170]_ = 25.03; *p* < 0.001; ηp^2^ = 0.280). Moreover, tibial loading during the follow-up assessment was significantly lower than that at post-intervention (mean difference = −3.91; F_[2,170]_ = 25.03; *p* < 0.001; ηp^2^ = 0.280) (see Figure 4).

### 3.5 Secondary outcomes

The two-way ANOVA revealed significant group × time interactions for ankle plantar/dorsiflexion angle (F_[2,170]_ = 4.71, *p* = 0.010, ηp^2^ = 0.036), estimated soleus force (F_[2,170]_ = 4.63, *p* = 0.011, ηp^2^ = 0.035), and TSK score (F_[2,170]_ = 12.23, *p* < 0.001, ηp^2^ = 0.088). Post hoc analyses showed that, in the EI group, ankle plantarflexion angle decreased significantly at both the post-intervention assessment (mean difference = −1.95; 95% CI: −3.26 to −0.65; F_[2,170]_ = 3.86; p = 0.008; ηp^2^ = 0.058) and the follow-up assessment (mean difference = −1.45; 95% CI: −2.57 to −0.33; F_[2,170]_ = 3.86; *p* = 0.050; ηp^2^ = 0.058), with no significant difference between the two time points (*p* = 0.488). Similarly, in the EI + PE group, ankle plantarflexion angle was significantly reduced at both the post-intervention assessment (mean difference = −2.35; 95% CI: −3.68 to −1.01; F_[2,170]_ = 21.69; *p* < 0.001; ηp^2^ = 0.252) and the follow-up assessment (mean difference = −4.19; 95% CI: −5.78 to −2.61; F_[2,170]_ = 21.69; *p* < 0.001; ηp^2^ = 0.252). Notably, values at follow-up were significantly lower than those at post-intervention (mean difference = −1.85; 95% CI: −2.78 to −0.91; F_[2,170]_ = 21.69; *p* = 0.004; ηp^2^ = 0.252).

For estimated soleus force, no significant changes were observed across baseline, post-intervention, and follow-up assessments in the EI group (*p* > 0.05). In contrast, within the EI + PE group, estimated soleus force was significantly lower than baseline at both the post-intervention assessment (mean difference = −0.23, 95% CI: −0.34 to −0.13; F[2,170] = 11.01; *p* = 0.003; ηp^2^ = 0.146) and the follow-up assessment (mean difference = −0.35, 95% CI: −0.51 to −0.20; F[2,170] = 11.01; *p* < 0.001; ηp^2^ = 0.146).

A similar pattern was observed for TSK score: the EI group showed no significant changes over time (*p* > 0.05), whereas the EI + PE group exhibited markedly lower TSK scores at both the post-intervention assessment (mean difference = −7.27; 95% CI: −9.70 to −4.84; F_[2,170]_ = 25.04; *p* < 0.001; ηp^2^ = 0.280) and the follow-up assessment (mean difference = −9.05; 95% CI: −11.72 to −6.37; F_[2,170]_ = 25.04; *p* < 0.001; ηp^2^ = 0.280) compared with baseline.

For variables without significant group × time interactions, *post hoc* analyses indicated that both the EI and EI + PE groups demonstrated significant improvements in estimated gluteus medius force following the 8-week intervention (mean difference = 0.03; EI group: 95% CI: −0.02 to 0.08; EI + PE group: 95% CI: 0.00 to 0.06; F_[2,170]_ = 2.48; *p* = 0.046; ηp^2^ = 0.019). In addition, compared with baseline, both groups exhibited significantly greater estimated tibialis anterior force at the post-intervention assessment (mean difference = 0.10; EI group: 95% CI: 0.02 to 0.18; EI + PE group: 95% CI: 0.03 to 0.15; F_[2,170]_ = 6.36; *p* = 0.001; ηp^2^ = 0.048) and at follow-up (mean difference = 0.09; F_[2,170]_ = 6.36; EI group: 95% CI: 0.01 to 0.14; EI + PE group: 95% CI: 0.04 to 0.16; *p* = 0.003; ηp^2^ = 0.048). It should be noted that the repeated *F* values derive from the same omnibus model and are therefore repeated across the corresponding pairwise contrasts (see Figure 4).

Secondary outcomes were analyzed with Benjamini–Hochberg FDR correction. In the EI + PE group, statistical testing indicated that ankle plantarflexion/dorsiflexion angle remained significantly reduced relative to baseline at both post-intervention (FDR-adjusted *p* = 0.007) and follow-up (FDR-adjusted *p* < 0.001), with a further significant reduction from post-intervention to follow-up (FDR-adjusted *p* = 0.002). TSK scores were also significantly lower than baseline at both post-intervention and follow-up (FDR-adjusted *p* < 0.001). In the EI group, the ankle plantarflexion/dorsiflexion angle remained significantly reduced post-intervention compared with baseline (FDR-adjusted *p* = 0.023), whereas the reduction at follow-up approached significance (FDR-adjusted *p* = 0.051). Additionally, following FDR correction, estimated soleus force in the EI + PE group was significantly reduced at follow-up relative to baseline (FDR-adjusted *p* = 0.026), with the post-intervention change approaching significance (FDR-adjusted *p* = 0.052). All other secondary outcomes did not reach statistical significance after FDR adjustment and should therefore be interpreted as exploratory.

## 4 Discussion

The present study aimed to investigate the practical effects of EI combined with PE on tibial loading and its associated risk factors in runners with MTSS. The intervention was designed not only to reduce tibial loading and improve maladaptive movement patterns, but also to enhance patients’ understanding of MTSS pathogenesis and pain-management strategies, thereby improving the overall quality of MTSS-related intervention. Our findings demonstrated that, after the 8-week intervention, both the EI + PE and EI groups exhibited significant reductions in tibial loading and ankle plantarflexion angle. However, during the 3-month follow-up, these improvements were maintained in the EI group, whereas the EI + PE group showed further reductions beyond the initial gains. Regarding model-derived muscle-force outcomes and psychological status, both groups exhibited significant increases in the estimated forces of the gluteus medius and tibialis anterior after the 8-week intervention. Notably, only the EI + PE group exhibited significant reductions in estimated soleus force, accompanied by a pronounced decrease in TSK scores. It should be noted that the findings of this study are applicable only to young male recreational runners with MTSS.

### 4.1 Primary outcome

This study found that both the EI and EI + PE interventions effectively reduced tibial loading during running; however, during follow-up, tibial loading remained relatively stable in the EI group, whereas the EI + PE group exhibited a further reduction. These findings align with previous research. For example, a systematic review reported that multimodal interventions incorporating PE not only enhance adherence to exercise-based therapy in patients with low back pain but also prolong the durability of treatment effects (Barbari et al., 2020). One plausible explanation is that individuals with MTSS often develop maladaptive movement patterns—such as excessive hip external rotation and increased ankle plantarflexion—due to long-standing pain (Hamstra-Wright et al., 2015). Prior studies have demonstrated that such altered movement patterns are closely associated with MTSS recurrence (Moen et al., 2012; Reinking et al., 2017). The central premise of PE is to facilitate patients’ understanding of the neurophysiological mechanisms of pain (Shala et al., 2021), thereby enabling them to consciously adjust their movement strategies during running. This cognitive reframing progressively promotes the adoption of more optimal movement patterns and mitigates the negative influence of pain on motor patterns (Shala et al., 2021). Importantly, these cognitive changes typically exert sustained effects, which may explain why the EI + PE group continued to show a progressive decline in tibial loading during follow-up. It should be noted that the potential sustained effects of EI + PE on tibial loading, as well as the associated mechanistic interpretations regarding its potentially greater durability, are speculative in nature and have not been empirically validated.

### 4.2 Secondary outcomes

Previous research has indicated that higher soleus activity during the push-off phase increases the risk of developing MTSS by approximately 5% (Naderi et al., 2020). In addition, a larger ankle plantarflexion angle has been associated with MTSS, with an effect coefficient of 0.7 (0.5–0.9) (Moen et al., 2012). Guided by these two established risk factors, the present study incorporated targeted EI and PE components. Specifically, the EI protocol emphasized strengthening the tibialis anterior to promote greater ankle dorsiflexion during running and reduce reliance on the soleus. In parallel, the PE component was designed to foster accurate movement perceptions, enabling individuals to consciously regulate ankle plantarflexion during running. After the 8-week intervention, both the EI and EI + PE groups exhibited significant increases in estimated tibialis anterior force and significant reductions in ankle plantarflexion angle. Because the EI intervention included targeted training to enhance tibialis anterior strength, both groups exhibited significant improvements in this muscle. Prior evidence suggests that greater active force generation by the tibialis anterior during the swing phase further contributes to accelerating the foot into dorsiflexion (Maharaj et al., 2019). Consequently, the EI intervention indirectly led to a reduction in ankle plantarflexion angle during running. At the 3-month follow-up, the EI group maintained the post-intervention plantarflexion angle, whereas the EI + PE group demonstrated a further reduction. Notably, only the EI + PE group showed a marked decline in estimated soleus force. This pattern may be attributed to the role of PE in promoting conscious dorsiflexion during running, thereby reducing excessive dependence on the plantarflexor muscles—particularly the soleus—during the loading and push-off phases, ultimately manifesting as a downregulation of soleus force (Cunnane et al., 2023). Such changes in plantarflexor recruitment appear to contribute to more sustained improvements in ankle plantarflexion angle. In contrast, the EI intervention alone is insufficient to induce changes in estimated soleus force through this mechanism, resulting in more transient effects on ankle plantarflexion. Considering that excessive soleus activation during push-off and increased ankle plantarflexion angle are key risk factors for MTSS (Moen et al., 2012; Naderi et al., 2020), these findings suggest that EI + PE may confer more durable protective benefits against the development of MTSS than EI alone. It should be noted that the mechanistic interpretations regarding the differential effects of the two interventions on the soleus muscle and ankle plantarflexion angle, as well as the proposed long-term preventive effects of the EI + PE group, are speculative in nature. These interpretations are based on existing literature and the specific characteristics of the present intervention, and have not been empirically validated by direct evidence from this study.

A larger hip external rotation angle is one of the key contributors to MTSS, primarily because increased external rotation alters the rotational alignment of the femur relative to the tibia. Such rotational deviations may propagate through the knee–tibia linkage, leading to excessive tibial internal rotation or abnormal bending loads, thereby elevating the risk of MTSS (Winkelmann et al., 2016). In the present study, although both the EI and EI + PE interventions effectively increased the model-estimated force of specific hip internal rotators—particularly the gluteus medius—during running in individuals with MTSS, neither intervention elicited meaningful changes in hip external rotation angles. This finding may be largely attributable to the fact that hip internal and external rotation are governed by the integrated action of multiple muscles. Consequently, strengthening a single internal rotator is insufficient to elicit a meaningful, system-level modification in hip internal rotation mechanics—that is, to effectively reduce excessive external rotation. Specifically, previous studies have indicated that the primary hip internal rotators include the tensor fasciae latae, gluteus medius, and gluteus minimus. However, conventional hip internal rotation exercises are generally unable to substantially enhance the strength of the tensor fasciae latae or the gluteus minimus. This is because the tensor fasciae latae contributes meaningfully to hip internal rotation only when the joint is positioned in a specific posture—namely, when hip flexion approaches 90° (Foss, 2024), which is the range in which it can be effectively targeted during training. Moreover, the gluteus minimus lies deep to the gluteus medius, and its relatively deep anatomical location limits its activation during conventional strengthening exercises (Foss, 2024). As a result, the internal rotation training in this study primarily influenced the gluteus medius, with relatively limited impact on other hip internal rotators. Importantly, reducing hip external rotation during running—that is, increasing hip internal rotation—relies on the coordinated activation of multiple muscle groups. Enhancing gluteus medius force alone, despite its role as one of the hip internal rotators, is insufficient to meaningfully decrease external rotation (or increase internal rotation) (Foss, 2024). Given that increased hip external rotation is a well-established risk factor for MTSS (Reinking et al., 2017), increases in model-estimated gluteus medius force alone are likely to exert only a limited effect on reducing recurrence risk or improving rehabilitation outcomes.

Regarding TSK scores, the present study found that a significant reduction occurred only in the EI + PE group. This improvement may be attributed to the cognitive-behavioral component of the EI + PE intervention, which helps reshape patients’ maladaptive cognitive responses to pain and facilitates the regulation of threat perception and avoidance behaviors (Leeuw et al., 2007). Such psychological adjustments may, in turn, promote the development of more appropriate load-management strategies and enhance patients’ motivation and compliance with subsequent exercise participation (Sheikhi et al., 2024), thereby interrupting the maladaptive “pain–fear–avoidance–functional decline” cycle (Sheikhi et al., 2024). Consequently, EI + PE demonstrated superior efficacy over EI alone in reducing TSK scores (Sheikhi et al., 2024). Given that elevated TSK scores may be linked to reduced muscle strength during exercise, impaired postural control, and diminished proprioception (Alshahrani and Reddy, 2022)—factors that may exacerbate existing MTSS risk—this finding carries important clinical implications. For example, Alshahrani and Reddy (2022) reported that fear of movement compromises proprioceptive accuracy during ankle plantarflexion, potentially altering plantarflexion angles during running, whereas Souza de Vasconcelos et al. (2023) noted that movement-related fear can also influence hip rotational patterns. Therefore, EI + PE-induced improvements in TSK may further reduce the potential risk of MTSS and highlight the greater clinical applicability of EI + PE in clinical intervention settings. It should be noted that the proposed mechanistic pathway whereby patient education may reduce kinesiophobia, subsequently improve motor control, and ultimately decrease tibial loading was inferred primarily from prior literature. The present study did not directly verify this pathway through mediation analysis or temporal-sequencing analysis. Therefore, this mechanism should be interpreted as a plausible explanatory hypothesis rather than as an empirically demonstrated causal pathway.

### 4.3 Clinical implications

The relatively prolonged effect of the EI + PE intervention on tibial loading, compared with the control group, suggests that education not only consolidates the short-term gains achieved through muscle-strengthening exercises but also facilitates more sustained and active modulation of abnormal ankle movement patterns and muscular activation strategies than that observed in the control condition. Therefore, intervention strategies for individuals with MTSS should prioritize the integration of EI and PE to establish a dual-component model that combines exercise training with cognitive education, thereby effectively reducing tibial loading and optimizing MTSS-related risk factors.

It is noteworthy, however, that the PE component demonstrated limited influence on hip joint movement patterns. Future intervention strategies may consider incorporating proximal joint–focused cognitive feedback modules while maintaining the existing PE framework. Examples include video-based feedback, movement imitation, or gait-training paradigms. These modalities may enhance patients’ awareness of necessary adjustments in hip kinematics during running, enabling them to actively regulate hip joint angles and associated muscular activation patterns—particularly hip external rotation and hip internal rotator strength, both strongly linked to MTSS. Such targeted modifications may further reduce tibial loading and enhance the overall efficacy of the intervention.

### 4.4 Limitations

Although this study provides meaningful clinical insights, several limitations should be acknowledged. First, the intervention period was relatively short, and the follow-up duration was limited to 3 months, which may not fully capture the long-term effectiveness of EI + PE in the rehabilitation of individuals with MTSS. Second, the optimal dosage and frequency of PE were not systematically examined, leaving the standardization and broader applicability of the intervention protocol to be further validated. In addition, this study employed a per-protocol analysis in which participants who did not complete the prescribed intervention protocol or failed to complete follow-up assessments were excluded. Although this approach facilitates the evaluation of the intervention’s effectiveness under conditions of full adherence, it may also introduce a degree of attrition bias due to sample selection and potentially compromise the internal validity and robustness of the findings. Therefore, when interpreting the results of this study, the potential impact of participant attrition and exclusion should be carefully considered. Although this trial was designed to emulate a pragmatic clinical intervention, the additional one-on-one education sessions provided to the EI + PE group may have elicited non-specific therapeutic effects—such as increased therapist attention, expectancy effects, strengthened therapeutic alliance, reassurance, or improved adherence—which should be acknowledged when interpreting the specific contribution of psychologically informed education. Furthermore, although the overall correlation coefficients and RMSE between EMG signals and OpenSim model activations fell within acceptable ranges, waveform alignment for certain muscles—such as the gluteus medius—still exhibited noticeable deviations. These discrepancies likely arise from factors including surface EMG susceptibility to cross-talk, motion artifacts of the skin, challenges in capturing deep muscle activity, and the reliance of OpenSim muscle activation estimates on model assumptions. Accordingly, these results should primarily be interpreted as supporting the plausibility of the model outputs rather than as a definitive, exact replication of *in vivo* muscle force generation. In addition, this study exclusively enrolled male recreational runners aged 18–30 years with a weekly running distance exceeding 20 km. Therefore, the findings may not be generalizable to female runners, adolescents, older runners, novice runners, military populations, or athletes with differing training loads. Finally, the study did not perform stratified analyses across different movement or sport-specific contexts, and thus the adaptability of EI + PE to varying activity scenarios remains unclear.

## 5 Conclusion

This study showed the comparative effects of the EI + PE intervention on MTSS-related biomechanical and psychological outcomes. Specifically, EI + PE was associated with reductions in tibial loading and ankle plantarflexion angle immediately after the intervention, and these parameters were further reduced at the three-month follow-up compared with the EI group. Moreover, the EI + PE intervention was associated with increased model-derived contributions of the gluteus medius and tibialis anterior, reduced model-derived contribution of the soleus, and substantially lower TSK scores; however, whether these changes translate into clinically meaningful benefits remains to be confirmed in future studies. It should be noted that the findings of this study are applicable only to young male recreational runners with MTSS, and caution is warranted when generalizing these results to other populations.

## Data Availability

The datasets analyzed during the current study are not publicly available because the project is ongoing, but they are available from the corresponding author upon reasonable request. Requests to access the datasets should be directed to ML, 848315077@qq.com.
