# Supplementary material for: Psychologically informed patient education combined with exercise intervention is associated with improvements in tibial loading regulation, joint kinematics, and kinesiophobia in runners with medial tibial stress syndrome: a randomized controlled trial
Source: Front Psychol. 2026 Jul 24;17:1880253. doi: 10.3389/fpsyg.2026.1880253 (PMC13447064; doi:10.3389/fpsyg.2026.1880253)
Supplement: Supplementary file 1 [file Table_1.docx]

Supplementary Material

# 1 Overall Design of the Intervention Protocol

The two intervention protocols implemented in this study were Exercise Intervention plus Patient Education (EI+PE) and Exercise Intervention (EI). The specific procedures and content of each protocol are described below. Both interventions were delivered by trained personnel, who introduced the intervention components to participants and provided individualized guidance for all training activities.

Participants in the EI group completed supervised, in-person exercise sessions three times per week, with each session consisting of four sets of prescribed exercises. Throughout the training process, correct movement execution was emphasized over the number of repetitions. In addition, each participant received a printed training manual outlining key technical points for resistance exercises and other relevant considerations. Participants were required to complete at least 16 sessions within the 8-week intervention period (i.e., 66% adherence to the prescribed program). No additional interventions or treatments were permitted during the study period.

In addition to the above exercise protocol, participants in the EI+PE group attended three individualized patient-education sessions, delivered by trained professionals. These sessions were conducted on a one-to-one basis during Weeks 1, 3, and 7, with each session lasting approximately 30–45 minutes.

2 **Exercise Intervention**

The exercise intervention in this study consisted of four resistance-training exercises, designed in accordance with the American College of Sports Medicine guidelines for strength training, with the primary objective of enhancing the strength of target muscle groups. All exercises incorporated both concentric and eccentric muscle contractions, and participants were instructed to perform each movement at a moderate velocity, with each concentric and eccentric phase lasting approximately 1 to 2 seconds. The recommended training intensity was 60% to 70% of one-repetition maximum (1RM), with 8 to 12 repetitions per set, for a total of three sets, and a rest interval of 1.5 to 2 minutes between sets.

## 2.1 Principles for Estimating RM

Elastic bands were used as the external load during training. Throughout the intervention, participants were required to reach muscular fatigue within 8 to 12 consecutive repetitions. If a participant was able to complete 14 or more repetitions without experiencing fatigue, the resistance was increased accordingly to ensure adherence to the principles of progressive loading.

## 2.2 Training Equipment

During the training sessions, resistance bands of varying tension levels or sandbags were used as the primary external loading tools. In addition, all participants were provided with standardized yoga mats to minimize discomfort or potential injury caused by localized pressure on hard surfaces, thereby improving overall training adherence.

## 2.3 Tibial Pain Monitoring

To ensure the scientific rigor of the training process, each participant’s subjective tibial pain level on the training day was assessed using a 0–10 cm Visual Analog Scale (VAS), where 0 indicates “no pain” and 10 represents “the worst imaginable pain.” Figure 1 illustrates the designated safe pain zone, acceptable pain zone, and high-risk pain zone. Participants completed VAS assessments before, during, and after each training session and recorded their scores in a training diary.

If a participant reported tibial pain of 6 or above before or during training, the exercise intensity was reduced accordingly—such as by lowering the resistance of the elastic band or decreasing the number of repetitions—to prevent symptom exacerbation. Additionally, both groups received explicit activity restrictions: aside from the prescribed training sessions, participants were instructed to avoid any daily activities or exercise forms that could provoke MTSS-related symptoms throughout the intervention period.

## 2.4 Intervention Content

The four exercises prescribed in the EI group primarily targeted the hip internal rotators and ankle dorsiflexors. These exercises were selected because they impose relatively low tibial loading, making them appropriate for individuals with MTSS. Furthermore, the exercises are simple to perform and allow for flexible adjustment and progression of training intensity.

**2.4.1 Warm-up Preparation**

Before each training session, participants completed 20 repetitions of seated hip internal rotation and toe raises as a warm-up. No external load was applied during this phase, which was intended to adequately activate the musculature surrounding the hip and ankle.

**2.4.2 Elastic-Band Hip Internal Rotation Training**

**Purpose:** This exercise aims to selectively strengthen the hip internal rotator muscles, primarily the gluteus minimus, gluteus medius, and tensor fasciae latae.

**Exercise Description:** Participants were instructed to adopt a seated or standing posture with an elastic band secured to the medial aspect of the ankle, while the opposite end was fixed to a stable support. The movement began with the participant slowly rotating the lower leg toward the body’s midline to perform hip internal rotation, followed by a controlled return to the starting position.

**Key Technical Points:** Throughout the exercise, participants were required to maintain pelvic and trunk stability, avoiding compensatory rotation or lateral flexion. Emphasis was placed on ensuring that the hip internal rotators served as the primary source of force generation, while minimizing excessive recruitment of the gluteus maximus or lumbar musculature.

**Training Progression:** Progressive loading was achieved by increasing the resistance level of the elastic band or lengthening the tension pathway, thereby allowing a gradual escalation in training demand.

**2.4.3 Quadruped Hip Internal Rotation Training**

**Purpose:** This exercise aims to enhance hip internal rotation control and promote coordinated stability between the core and lower limbs.

**Exercise Description:** Participants assumed a quadruped position with the knees aligned at hip width. At the start of the movement, one knee was maintained in approximately 90° of flexion while the lower leg was internally rotated toward the body’s midline, followed by a slow and controlled return to the neutral position.

**Key Technical Points:** Throughout the exercise, participants were instructed to maintain spinal and pelvic stability, avoiding trunk sway or compensatory lumbar motion.

**Training Progression:** Training load was progressively increased by attaching an elastic band to the ankle to introduce additional resistance.

**2.4.4 Standing Toe Raises**

**Purpose:** This exercise is designed to systematically strengthen the ankle dorsiflexor muscle group and improve both force-generating capacity and neuromuscular control.

**Exercise Description:** Participants assumed a standing position with both feet placed parallel on the floor. The movement began by lifting the forefoot and toes off the ground to achieve maximal dorsiflexion while maintaining heel contact, followed by a slow and controlled return to the starting position.

**Key Technical Points:** Participants were instructed to keep the torso upright and maintain postural stability, avoiding excessive knee extension or compensatory motion at the hip.

**Training Progression:** To enhance training load, participants could use a wall for balance support, progressively increase the number of repetitions, or add external resistance by attaching a weight to the dorsal aspect of the foot.

**2.4.5 Seated Ankle Dorsiflexion Training**

**Purpose:** This exercise aims to strengthen the ankle dorsiflexor musculature and improve both its force-production capacity and neuromuscular control.

**Exercise Description:** Participants were seated with both feet placed flat on the floor. The movement began by lifting the forefoot off the ground while maintaining stable heel contact, achieving maximal dorsiflexion before slowly returning to the starting position.

**Key Technical Points:** Participants were instructed to perform the movement with smooth and controlled motion, avoiding unintended knee or hip involvement and emphasizing activation of the tibialis anterior.

**Training Progression:** Progressive overload was introduced by gradually increasing the number of repetitions or adding light external resistance to the dorsal aspect of the foot.

3 **Patient Education**

## 3.1 Why Does MTSS Occur?

Under normal conditions, the stability of the lower-limb kinetic chain relies on the coordinated motion of the hip, knee, and ankle joints. Among these structures, hip rotation and ankle plantarflexion play pivotal roles in regulating lower-limb alignment, attenuating impact forces, and distributing mechanical stress during locomotion.

However, excessive external rotation of the hip can give rise to compensatory movement patterns during the stance phase, such as increased tibial internal rotation. This altered biomechanical state concentrates ground reaction forces along the medial border of the tibia, thereby elevating the mechanical load on the periosteum and surrounding soft tissues. At the same time, excessive ankle plantarflexion markedly increases the traction forces exerted by the posterior compartment muscles—particularly the gastrocnemius and soleus—on the medial aspect of the tibia during running. This added tension further exacerbates local stress accumulation and intensifies pain.

In addition, persistent pain often induces fear of movement among individuals with MTSS. Such fear can drive maladaptive gait modifications, including exaggerated ankle plantarflexion or altered hip rotation patterns, which in turn amplify the very biomechanical risk factors that contribute to MTSS onset and recurrence.

Collectively, the coupled effects of hip external rotation and ankle plantarflexion expose the medial tibia to repeated high-intensity mechanical loading, predisposing the periosteum to microtrauma and inflammatory responses. Moreover, movement-related fear further perpetuates these biomechanical deviations, creating a self-reinforcing cycle that increases the likelihood of symptom persistence or recurrence. These combined mechanisms are widely recognized as the central pathological basis of MTSS.

## 3.2 How Can Movement Patterns Be Improved?

During running, particular attention should be given to maintaining stable lower-limb alignment during the stance phase to reduce the risk of MTSS and minimize localized stress concentration. First, excessive ankle plantarflexion at initial contact should be avoided; keeping the foot oriented straightforward helps reduce the traction imposed by the posterior compartment muscles on the medial aspect of the tibia. Second, maintaining rotational stability of the hip in the transverse plane is essential. Excessive hip external rotation can alter lower-limb alignment, thereby increasing compensatory tibial stress and elevating the mechanical load on the medial tibial border.

## 3.3 How to Manage Pain and Reduce Training Load?

MTSS-related symptoms commonly arise during physical activity, leading many individuals to cease exercise entirely to avoid worsening pain. However, recent evidence suggests that maintaining low-intensity activity within a tolerable pain range can facilitate the central nervous system’s ability to perceive and integrate somatosensory feedback. This process helps optimize movement patterns, regulate mechanical loading more effectively, and ultimately supports functional recovery and symptom alleviation. For instance, if MTSS symptoms emerge during running, patients may adopt an interval strategy that alternates between running and walking while monitoring changes in pain. This approach reduces joint mechanical loading while preserving lower-limb muscle activation, thereby preventing functional decline associated with complete rest.

As a general guideline, physical activity may be continued as long as pain does not exceed 5/10 on the VAS and symptoms do not worsen the following day. Additionally, delayed-onset muscle soreness after training is considered a normal physiological response and does not warrant concern.

## 3.4 How to Safely Return to Sport?

When resuming running after a period of rest, a gradual and structured reintroduction of training load is essential. For example, if an individual previously engaged in one-hour running sessions, the initial phase of return should be limited to warm-up activities or 15 minutes of low-intensity exercise. The patient should then assess pain intensity and general physical response on the following day. If symptoms do not worsen, the duration of the next session may be increased to 30 minutes. After two consecutive weeks of completing the full training routine with no pain or only minimal discomfort, the patient may progressively transition back to full training and eventually resume competitive participation.

Conversely, a rapid or excessive increase in training load during the return-to-sport process may exceed the body’s tolerance and consequently aggravate symptoms. Therefore, carefully regulating both the rate and magnitude of load progression is essential for ensuring the sustainability and effectiveness of rehabilitation training.

## 3.5 Educational Feedback

At the end of each patient education session, individuals are encouraged to raise questions or express concerns based on their personal experiences. The intervention personnel should provide thorough and professionally grounded explanations, ensuring that all information is accurate, individualized, and clinically relevant. This process helps patients gain a clearer understanding of their condition, enhances their self-management capability, and improves adherence to the prescribed treatment plan.

4 **Supplementary Results**

## 4.1 Between-group differences in primary and secondary outcomes

Between-group comparisons at the post-intervention and follow-up assessments showed that, immediately after the intervention, the EI+PE group had significantly lower TSK scores than the EI group (mean difference = -7.52, 95% CI: -9.68 to -5.35, P < 0.001). At follow-up, the EI+PE group demonstrated significantly lower tibial load (mean difference = -3.61, 95% CI: -5.26 to -1.96, P < 0.001), ankle plantar/dorsiflexion angle (mean difference = -2.81, 95% CI: -4.19 to -1.43, P < 0.001), soleus force (mean difference = -0.24, 95% CI: -0.38 to -0.10, P < 0.001), and TSK scores (mean difference = -8.68, 95% CI: -11.30 to -6.06, P < 0.001) than the EI group. No significant between-group differences were observed for the remaining secondary outcomes at either assessment time point (all P > 0.05).

## 4.2 Analysis of the Effect of Running Speed on Tibial Loading: Covariate Analysis

Because tibial loading is sensitive to running speed, an additional covariate-adjusted analysis was conducted with running speed included as a covariate in the model. Specifically, tibial loading was specified as the dependent variable, with Time, the Group × Time interaction, and running speed entered as fixed effects, and participant identity included to account for the repeated-measures structure. After adjustment for running speed, the Group × Time interaction for tibial loading remained statistically significant, F(2, 169) = 5.402, P = 0.005, partial η² = 0.060. The covariate effect of running speed itself was not statistically significant in this model, β = 0.935, t = 0.431, P = 0.667.

## 4.3 OpenSim Validation

Validation results demonstrated that the sagittal-plane marker errors at the hip, knee, and ankle joints were all below 5°, indicating satisfactory inverse kinematics fitting (Table S1). The joint-angle RMSE values exhibited minimal variation, suggesting that the model reasonably reproduced the joint motion trajectories (Table S1). Furthermore, examination of the RRA residuals revealed that both force and moment values, after normalization, remained within reasonable ranges, indicating well-controlled residuals. Concerning CMC tracking errors, the results showed hip, knee, and ankle RMSE ≤ 2.11°, reflecting a close correspondence between muscle-driven simulations and target motions. Regarding EMG–model activation agreement, correlation coefficients ranged from 0.73 to 0.93, RMSE values from 0.06 to 0.22, and temporal differences were ≤ 0.03 s. These metrics fall within previously reported EMG–OpenSim matching ranges, confirming that the model-derived activations reasonably capture experimentally measured muscle activity.

Table S1. Sagittal-plane IK marker errors and joint-angle RMSE

| **Metric** | **Mean ± SD (°)** | **Estimated RMSE (°)** |
| --- | --- | --- |
| Hip sagittal-plane joint-angle error | 1.21 ± 1.01 | 1.58 |
| Knee sagittal-plane joint-angle error | 1.32 ± 1.01 | 1.66 |
| Ankle sagittal-plane joint-angle error | 1.28 ± 0.95 | 1.59 |

Table S2. Body-mass- and height-normalized RRA residuals

| **Residual component** | **Normalized value** |
| --- | --- |
| FX (BW) | 0.167 |
| FY (BW) | 0.200 |
| FZ (BW) | 0.155 |
| MX (BW·m) | 0.399 |
| MY (BW·m) | 0.370 |
| MZ (BW·m) | 0.425 |

Table S3. CMC sagittal-plane tracking errors

| **Tracked coordinate** | **Plane** | **CMC tracking error, RMSE (rad)** | **CMC tracking error, RMSE (°)** |
| --- | --- | --- | --- |
| Hip flexion/extension | Sagittal | 0.037 | 2.11 |
| Knee flexion/extension | Sagittal | 0.029 | 1.64 |
| Ankle plantarflexion/dorsiflexion | Sagittal | 0.024 | 1.40 |

Table S4. EMG–OpenSim activation agreement

| **Muscle** | **Pearson correlation coefficient (r)** | **EMG–activation RMSE** | **Peak timing difference (s)** |
| --- | --- | --- | --- |
| Tibialis anterior | 0.731 | 0.081 | 0.018 |
| Tensor fasciae latae | 0.933 | 0.081 | 0.021 |
| Soleus | 0.856 | 0.062 | 0.001 |
| Gluteus medius | 0.810 | 0.223 | 0.029 |
